# Nanoporous Structure of Bone Matrix at Osteoporosis from Data of Atomic Force Microscopy and IR Spectroscopy

**DOI:** 10.4061/2011/162041

**Published:** 2011-05-03

**Authors:** A. A. Gaidash, L. N. Sinitsa, O. A. Babenko, A. A. Lugovskoy

**Affiliations:** ^1^Department of Pharmacology, Siberian Federal Medical University, Krasnoyarsk 660041, Russia; ^2^Laboratory of Molecular Spectroscopy, V.E. Zuev Institute of Atmospheric Optics SB RAS, Tomsk 634021, Russia

## Abstract

It was found that in an osteoporotic bone the fraction of nanosized pores decreases, the mineral phase amorphizes, hydrated shells around mineralized particles of the bone matrix thicken, and adhesion forces increase. This contributes to the formation of water clusters similar to bulk water clusters compared to the healthy bone tissue and leads to the accumulation of more viscous liquid with increased intermolecular interaction forces in the pores of the bone matrix. Given this, the rates of chemical reactions proceeding in the water phase of ultrathin channels of general parts of collagen fibrils decrease. Ultimately, nanopores of collagen-apatite interfaces lose, to a certain extent, the capability of catalyzing the hydroxyapatite crystallization.

## 1. Introduction

An urgent problem of osteoporosis is the low efficiency of its correction with pharmaceuticals, which does not exceed 10% in mineral density for all the existing types of medical preparations [1]. This implies that a principally new paradigm is necessary to understand and correct osteoporosis. It should not only rely on technologies of intervention and optimization of calcium intake or stimulation of osteoblasts, but on getting a better insight into the causes of osteoporosis based on the ideas of hydroxyapatite (HAP) crystallization rates. The HAP crystallization mechanisms are due to, in this way or another, the mass transfer phenomena where water circulating in ultrathin channels of the bone matrix plays an important role. Most of these channels are located in collagen-apatite interfaces, whose structural state at osteoporosis is poorly studied. Structural properties of nanopores of the bone matrix and the water circulating in them and their role in hydroxyapatite crystallization mechanisms have not been understood so far.

It is known that one of the pathogenetic factors of osteoporosis is a dyshormonal lysis of collagen fibrils. When this is the case, the volume of the organic matrix, where crystallization of the HAP occurs, decreases, it is reasonable to assume that lysis begins with dissolution of thin fibrils of protocollagen, and vacant spaces are filled with bulk water. Thus, the swelling inside the fibrils and subsequent demineralization of the collagen fibrils might be one of early stages of osteoporosis.

The aim of this work is to study the fine structure of the bone matrix and the water clustering in it at osteoporosis by the methods of dynamic infrared spectroscopy and atomic force microscopy (AFM).

## 2. Materials and Method

The experiments were conducted with Wistar rats having a body weight of 250 g. Osteoporosis was modeled by bilateral testiculectomy. The length of the experiment was 3 months. Experimental groups contained only five rats (reference and osteoporotic), since the focus in this study was on structural properties of the bone matrix rather than with respect to different populations. Moreover, this was dictated by the ethical considerations. The test animals were kept and then euthanized in accordance with the World Medical Association Declaration of Helsinki, 1989, the RF Order “Animal Protection from Abuse” (1.01.1997), and the Order of the RF Ministry of Health N267 (19.06.2003).

### 2.1. Atomic-Force Microscopy

The structural properties of the healthy and osteoporotic bones were studied by the atomic-force microscopy. In atomic-force microscopy [2], the relief of the sample is investigated by a movable probe-cantilever. The cantilever is a silicon plate measuring size 3 × 1.5 × 0.3 mm with a sharp needle located at the end of the console. When the cantilever approached to the sample as close as several angstroms, repulsive forces predominate on the tip of the cantilever, while at larger distances attractive forces. As a result, the cantilever bends towards the sample or, conversely, unbends from the surface until the forces of elastic deformation of the sample and the probe become equal. The height of the cantilever—the distance from the tip of the needle to base is in the range from 3–6 microns. The radius of curvature of the tip is close to the X-ray scattering wavelength (1 to 50 nm). This allows us to obtain images that are consistent with the data of X-ray analysis. In a contact mode, when the probe tip touches the sample, a low stiffness cantilever is used, which increases sensitivity and softens the effect of mechanical action on the sample. In contact AFM-microscopy, we can determine the parameters of the local force interaction between the probe and sample surfaces, the force of friction, the local stiffness, and the spreading resistance. The contact atomic-force microscopy provides stable good quality images at high scan rate (up to 500 kHz) and allows us to estimate the level of hydrophilicity of small structural unit. For atomic-force microscopy use was made of a NTEGRA Prima probe nanolaboratory. The conditions of AFM scanning were the following: sample scanning was performed in air in the contact mode by CSG10 silicon cantilever with a radius of curvature of 10 nm at a lateral resolution of 0.24 nm. 

Linear dimensions of diameter and the domains of collagen fibrils, as well as other particles in the bone matrix, were determined by an Image Analysis module of the nanolaboratory. One sample from the diaphysis of the femur was taken for morphological studies of a rat. Statistical characteristics of the average group values were determined from 50 measurements. The diameter of collagen fibrils was determined from 50 segments, the values of adhesion forces from the hysteresis lines of “tap-drive” curves of the cantilever. The statistical characteristics for each group were calculated by standard formulas for the 95% confidence interval. The reliability of intergroup variation was calculated by the Student's *t*-test (*t*_st_), after sampling for normality of distribution.

Quantitative analysis of the phase composition of the mineral bone components was performed in a “Shimadzu XRD-6000” X-ray diffractometer in Bragg-Brentano geometry using a focusing pyrographite monochromator in the scattered beam. Processing of the diffraction patterns of polycrystalline samples was carried out using an ICDD database diffraction data and the full-profile analysis software code HOWDER CELL 2.4.

### 2.2. Dynamic IR-Spectroscopy

Earlier [3, 4], it was shown that water, specifically its IR-absorption, is an effective probe of the properties of nanoporous structure of the object under study. The shape of the absorption band of water in pores is determined by the degree of bonding of water molecules (its cluster composition); and the cluster structure of water in the pores, in turn, is determined by the pore size. The probing properties of water were generally studied at around 3400 cm^−1^ (region of stretching vibration) [3]. Since variation of this band with pore parameters is very small, we use region of combination band, 5400 cm^−1^, where this variation is much stronger. 

The absorption spectrum of water was registered using a Bruker IFS-125M Fourier spectrometer. The spectral resolution of the spectrometer was varied from 1 to 20 cm^−1^. In this paper, we used dynamic registration of the absorption spectrum of water in the sample. First, we recorded the absorption spectrum of the sample filled with water, then the water was removed by the vacuum pumping and the sample spectrum was recorded at 10–20 min intervals under constant vacuum pumping (*I*_1_ ⋯ *I*_10_). The signal (*I*_*i*_) contains the absorption of dry samples (with the absorption coefficient *K* (sample)) and the absorption coefficient of water in the sample *K* (water), so that the total absorption coefficient is *K*_*i*_ = *K*_*i*_ (sample) + *K*_*i*_ (water). 

The ratio of the light intensity passed through the sample with water (*I*_*i*_) to the light intensity passed through the dry sample at the last moment of pumping (*I*_0_) gives the absorption spectrum of the water contained in the sample at the point of measurement. 

The ratio of the light intensity passed through the sample with water (*I*_*i*_) to the light intensity passed through the sample at the next point of time (*I*_*i*+1_) gives the absorption spectrum of the portion of water removed from the sample between the measurements:

(1) $$A_{i}^{*}\left(\nu \right)=1-\frac{I_{i}}{I_{i+1}}=1-\exp\;\left[-\left(\Delta K_{i}\;\left(\text{water}\right)\;L\right)\right],$$

where Δ*K*_*i*_ (water) is the absorption coefficient of the portion of water evacuated from the sample of length *L* during the time Δ*t*_*i*_. The value of the microportion absorption is very small (1 − *I*_*i*_/*I*_*i*+1_ < 10%) and goes down to zero within 60–90 minutes of the vacuum pumping. In this case, the spectrum becomes more variable and hence, more informative. 

First, we examined the spectra of water in the SiO_2_ samples with pores in the diameter range of 2.6 nm–50 nm which allowed us to determine a quantitative relationship between the spectra and the size of nanoporous structure. We have found the following.

**Figure 1:**
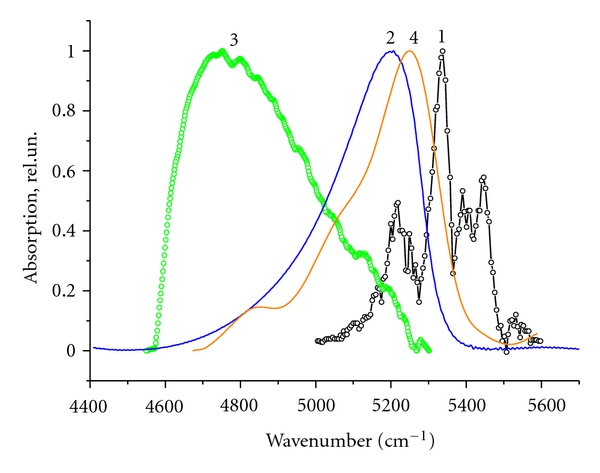
The absorption spectra in the range of 4500–5800 cm^−1^ of water monomer (1), bulk water (2), and water clusters in SiO_2_ with pores diameter of 12 nm (3) and 50 nm (4).

**Figure 2:**
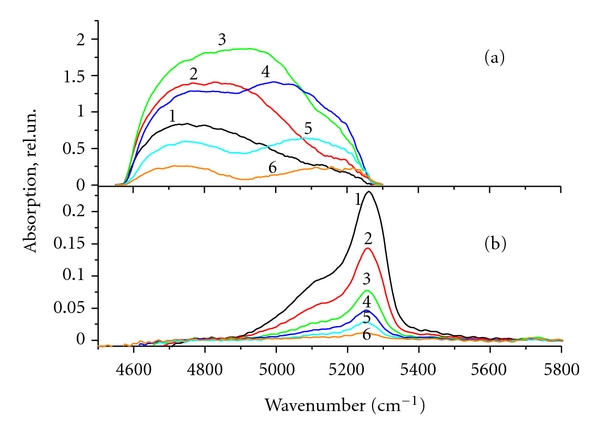
The absorption spectra of water microportions removed from SiO_2_ with pores diameter of 12 nm (a) and 50 nm (b) in the 4500–5800 cm^−1^ range, recorded at 10 (1), 20 (2), 30 (3), 40 (4), 50 (5), and 60 (6) min after the start of vacuum pumping.

(1) The spectra of water in the SiO_2_ samples with pores of different diameter (2.6 nm, 6.4 nm, 11.8 nm, and 50 nm) in the 4600–5400 cm^−1^ region consist of several subbands, which are well resolved (the interval between the subband peaks reaches 580 cm^−1^ for pores of the same size), and the band maxima for different size pores are located at different frequencies. The situation differs drastically from the 3000–3600 cm^−1^ region where the bands of bulk water and water in the SiO_2_ samples have a maximum at a frequency of 3430 cm^−1^ and its band contour is only slightly changed with variation in the pore size.

(2) When the pore diameter is increased from 2.6 nm to 6.4 nm, the absorption bands of clusters in this frequency range are shifted by 200 cm^−1^ from the water monomer band towards low frequencies, which indicates an increase in the hydrogen bond strength of confined water with an increase in the pore diameter. When the latter is as large as 12 nm the band center reaches the frequency of 4700 cm^−1^, which corresponds to the absorption of ice-like water. With a further increase in the pore diameter, the interaction of water clusters with sample walls decreases, the water acquires the properties of bulk water, and the maximum of the water cluster absorption is shifted to the absorption band of bulk water at 5200 cm^−1^.  Figure 1 shows the absorption spectra of water clusters in the SiO_2_ samples with the pore diameters of 11.8 and 50 nm.

(3) The spectrum recorded during water pumping is extremely variable in time, and the cluster dynamics in large pores (50 nm) differs greatly from that in middle-size pores (12 nm) (Figure 2). All types of water clusters are removed from large pores uniformly, and the absorption band has a sharp peak, which corresponds to weakly bound water clusters, while in the case of middle-size pores (12 nm), the water clusters associated with strong hydrogen bonds are removed from the sample at the beginning of the vacuum pumping, and the loosely coupled clusters are removed later. Moreover, the absorption band has several peaks corresponding to strongly and weakly bound water clusters. In the course of pumping out, of water undergoes restructuring and, hence, the peaks in the absorption spectrum are redistributed. This is due to the fact that the filling factor in large pores is not important, because the interaction of water with the walls takes place only within a narrow layer of water. Therefore, all water clusters in the large pores would be removed simultaneously, and the evacuation rate of the molecules from the pores would decay exponentially in accordance with the solution of the diffusion equation.

## 3. Results

### 3.1. Atomic-Force Microscopy

**Figure 3:**
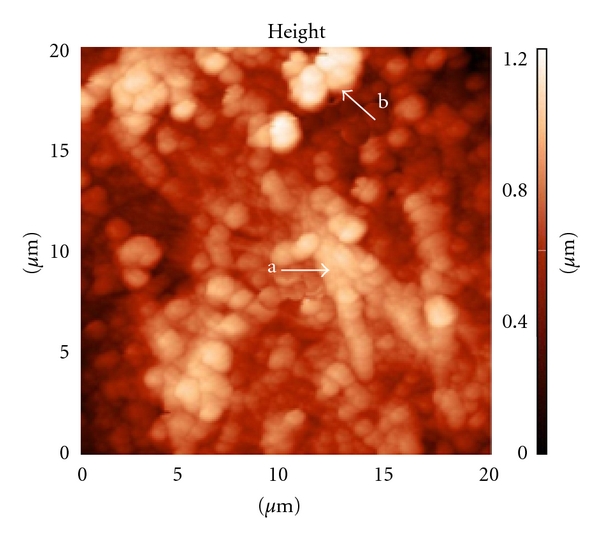
Granular structure of the bone matrix of the femur in healthy rats. Ordered arrangement of granular structures repeats the form of collagen fibrils. AFM scan size of 10 microns. Grain of regular structure (arrow a). Grain of irregular structure (arrow b).

According to the AFM data, a matrix of tubular bones of a healthy rat has a granular structure.  Figure 3 shows the AFM-image of the fine structure of bone matrix of healthy rats at a spatial resolution of 1 nm. It is seen from the figure that the matrix is formed by regularly arranged grains-spheres. The brightness of grains depends on the depth position relative to the surface of the cantilever. From the morphology, each “grain” is an extended section of the general part of the collagen fibrils (GPCF) (arrow a). This extension is due to hydroxyapatite crystals accumulated on the longer axis of collagen fibrils and selectively deposited only in those parts of domains. The grains, located in the fibrils, are isomorphic and have a clear outline. This indicates that the grains were formed with hydroxyapatite of the same well-crystallized phase. However, in the bone matrix of healthy rats we observed grains of irregular structure (arrow b). Central parts of the grains are still dense, while less dense zones are found on the periphery. This changes each of these grains into “core-shell” structure. We believe that “core-shell” grains can be formed by destroyed fragments of mineralized collagen fibrils that contain not only well-crystallized, but alsoamorphous hydroxyapatite. Therefore, appearance of the destroyed fragments of collagen fibrils containing amorphous HAP may be due to processes of the bone matrix self-renewal. In the future, these fragments could be utilized by osteoclasts. At a higher resolution, one observes crystals lying on the surface of the spheroids. The crystals form a characteristic pattern of D periods of collagen fibrils, and, as a result, GPCF look like oblate spheres (OS). According to the data of X-ray diffractometry, the crystals are represented by hydroxyapatite with a size of 21–25 nm, 80% of which have a hexagonal form and 20% of which have a monoclinic form.

Structural properties of bone matrix were studied morphometric and are listed in Table 1. According to statistical analysis, the diameter of OS along collagen fibrils achieves 848 (64) nm. General parts of D periods rise above the surface by 295 ± 27) nm and the thickness of granular-fibrillar structures is 1362 ± 121 nm. By the data of local adhesiotomy, the level of adhesion forces in the region of general parts of collagen fibrils varies within 18.6 nN, while in friable parts of collagen fibrils (FPCF) it achieves 26.4 nN. These levels of the adhesion forces indicate that the structure of GPCF compared to narrow parts is formed by the less wettable phase. In other words, the hydrophobic compartment (general part of a D period) and the hydrophilic compartment (friable part of D periods) can be separated in the structure of collagen fibrils of healthy rats. This confirms the previously discovered bimodal distribution of water in collagen interfaces [5, 6].

According to this, in GPCF or electron-dense parts (with a length of 0.46 D), the cross channels formed by parallel tropocollagen fibrils vary within 0.7–1.0 nm, while in electron-friable parts (with a length of 0.54 D) the diameter of ultranarrow slits achieves ~3.5–5.0 nm [7], and they are filled with water, undergoing a phase transition, similar to freezing of bulk water. Note that due to unclear mechanisms, the crystallization of precursor apatite occurs in ultrathin channels which are the main sections of the collagen domain.

**Figure 4:**
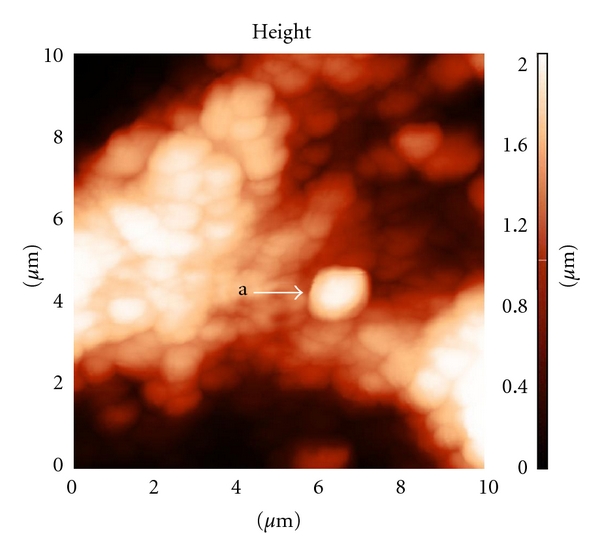
The disintegration of granular structure of the bone matrix of rat femur with osteoporosis. The arrow indicates the presence of free particle with signs of amorphization. AFM scan size of 10 microns.

Figure 4 shows a matrix of tubular bones of the osteoporotic rats. As follows from the figure, the disintegration of mineral-collagen spheroids is observed in the bone matrix of osteoporotic rats. This is manifested primarily in the form of a more chaotic arrangement of “grains”. Released from the collagen fibrils grains are Dual view (arrow a). Morphometric parameters change as well. In particular, the OS-diameter decreases down to 391 nm and the height of the convexity of GPCF decreases to 162 ± 32 nm. This change in the geometric parameters can be explained by the decrease in the size of hydroxyapatite crystals. The same is also indicated by the data of X-ray diffractometry, according to which the fraction of the monoclinic modification of HAP Ca_10_(PO_4_)_6_(OH)_2_ increases up to 12.0 vol.% in osteoporotic bone samples. Spheroids clearly experience the longitudinal compression as well. The compression energy is likely sufficient for the complete extrusion of spheroids out of the structure of collagen fibrils. This is also confirmed by the data of the morphological investigation obtained from the AFM scanning. Morphologically, the released spheroids take the shape of particles with blurred, sometimes two-profile edges (Figure 2). Released particles fill resorption lacunas and the microchannels. Taking into account this data, we can describe the domain destruction of collagen fibrils, which occurs in the direction of increasing the electron-friable sections. According to the data of local force microscopy, the adhesion forces increase nearly twice all over the length of mineral-collagen spheres, namely, up to 38 nN in GPCF and up to 52 nN in friable parts. Thus, at osteoporosis the physical properties of the both domains of collagen fibrils are smoothed by the wettability criterion in the sense that the volume of viscous parts increases, while the volume of apatite-synthesizing parts, to the contrary, decreases. Analogous changes in the level of adhesion forces are also observed beyond mineralized collagen fibrils, namely, in the so-called structureless parts of the bone matrix (from 18.6 nN in the control to 38 nN). The increase of adhesion forces in all structures of the bone matrix indicates that the more viscous liquid is accumulated in the compact bone substance at osteoporosis. In this connection, it should be noted that according to the data of X-ray diffractometry the fraction of the amorphous phase of HAP increases sharply in an osteoporotic bone: up to 36 vol.%. The measured parameters of the healthy and osteoporotic bones are presented in Table 1.

As follows from Table 1, the diameter of granules in bones with osteoporosis is almost two times less than in control. Also in the osteoporotic bone, a significant increase in adhesion strength occurs at the surface of collagen fibrils. The principally important fact is that features of amorphization are also observed in the mineral phase associated with collagen fibrils. The amorphization of the mineral phase is unambiguously indicative of the increase in the thickness of the hydrate shell around HAP crystals. It should be noted that the increase in the thickness of the hydrate shell around HAP crystals, whose diameter along the short axis varies within 1–1.5 nm, occurs in ultra thin channels of collagen fibrils, whose cross-section does not exceed 2 nm. Thus, it is reasonable to assume that this exerts the disjoining repulsive effect on nanotubules walls. In our opinion, this is the driving force disjoining the walls of the channels of collagen fibrils at osteoporosis. The results of local adhesion study clearly show that the fine structure of collagen fibrils accumulates water.

The expanding of ultra thin channels of the collagen interfaces can be examined by water spectra registration.

### 3.2. Dynamic IR-Spectroscopy

**Figure 5:**
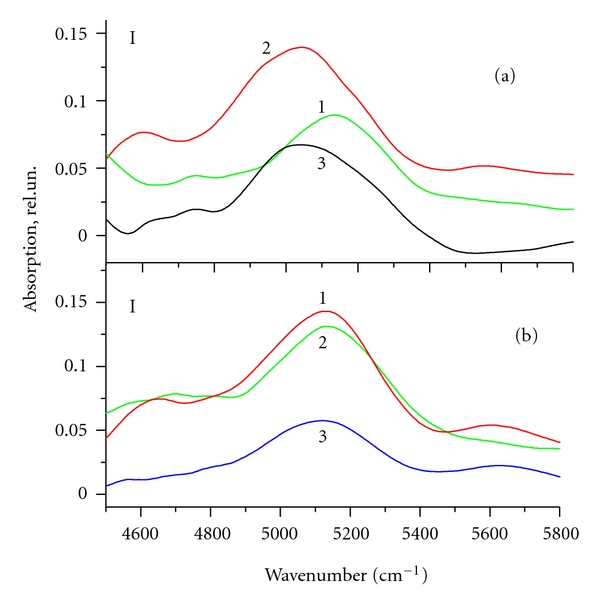
The absorption spectra of H_2_O in bone of healthy (a) and osteoporotic (b) rats in the 4570–5300 cm^−1^, recorded in 10 (1), 20 (2), 30 (3), minutes after the start of vacuum pumping. The error bars represent the corresponding 95% confidence interval.

The water was introduced in the sample keeping bones in water at 35°C during 24 hours. Absorption spectra of water in the bones were recorded using dynamic registration when the water was removed by the vacuum pumping and the sample's spectrum was recorded at 10–20 min intervals at constant vacuum. Spectra of water micro portions *A*_i_*(*ν*) registered in the vacuum pumping are shown in Figure 5. The centers of the water absorption bands for the osteoporotic bones are situated near 5150 cm^−1^ and those of healthy bones are shifted to the low frequencies of 5000–5150 cm^−1^. It is seen from the figure that the time behavior of water clusters for pores of healthy and osteoporotic bones differs significantly. 

The spectrum of water microportions in the healthy bones recorded during the pumping is variable in time. At the initial stage of vacuum pumping, the combination band in the healthy bones has a maximum at a frequency of 5160 cm^−1^. In the pumping-out process, the band contour changed resulting in the redistribution of subband intensities. The absorption center of gravity is shifted up to lower frequencies. The molecules which are bound by weak hydrogen bonds are removed from the nanopores at the initial moment of the pumping (see curve 1 in Figure 5(a)). The removal of strongly bound water molecules with maximum absorption at a frequency of 5000 cm^−1^ occurs at the end of pumping (see curve 3 in Figure 5(a)). As seen from Figure  5(b), the maximum of the water absorption band (5150 cm^−1^), in the osteoporotic bone, is situated closer to absorption of bulk water; in the spectrum there are no bands characterizing ice-like water clusters; there is no variability during water evacuation from the bones.

The area under the absorption curve is proportional to the number of molecules departing from the sample during the interval between measurements (10 min), that is, to the evacuation rate of the molecules. The area under the absorption curves 1, 2, 3 in Figure  5(b) in the osteoporotic bones decays exponentially with time. Figure  5(a) (curves 1, 2, 3) shows that the rate of water removal from the healthy bone does not obey the exponential law and does not behave no monotonically. The rate of water removal even increased in the middle of the evacuation process. Analysis shows that the water was removed from the osteoporotic bone samples with a much lower rate than from healthy bones, which indicates an increase in its viscosity.

All these three factors (the band centers, its variability during vacuum pumping, and evacuation rate) clearly indicate that healthy bones have distinct nanoporous structures whose dimensions are close to 10 nm. At the same time, these three factors show that the structure of osteoporotic bone is broken; this is manifested in the increase of collagen fibrils ultra thin channels in osteoporotic bones in comparison with healthy ones. The measured parameters of the water absorption bands for healthy and osteoporotic bones are presented in Table 2.

## 4. Discussion

It is known that the crystallization of precursor apatite occurs in ultrathin sections of the main channels of collagenous domains, which have a smaller diameter and, as we have shown greater hydrophobicity compared to the friable parts. There is no clarity in understanding the mechanisms of this selectivity location crystallization of apatite. We believe that a key factor may be differences in the rates of chemical reactions that take place in the aqueous phase of GPCF and FPCF. There are appropriate references in the literature that in ultrathin channels whose walls have hydrophilic surface, water is more viscous compared to water, located in ultrathin channels whose walls are more hydrophobic surface [8]. High viscosity medium, located in FPCF, suggests a lower rate of chemical reactions. Then it becomes a clear biological meaning of the organization of ultrathin channels of (GPCF). With a more hydrophobic surface, the water becomes less viscous and the rate of chemical reactions will be significantly higher than that in ultrathin channels of FPCF. Ultrathin channels of GPCF in such a situation actually perform the role of nanocatalysts, accelerating the crystallization of hydroxyapatite. What happens in osteoporosis? As follows from data of dynamic infrared spectroscopy of bone matrix in osteoporosis the relative volume of pores of smaller diameter decreases and the pores of large size appear. The water was removed from the osteoporotic bone samples with a much lower rate, which indicates an increase in its viscosity. Thus, a key factor in osteoporosis is the transformation of ultrathin nanopore channels of collagen fibrils in a more broad. What enhances the nanopores? It was shown above according to the AFM scanning that there is a pronounced amorphization of the mineral phase, which, of course, is accompanied by an increase in the thickness of the hydration shell around the nanocrystal hydroxyapatite. If we consider that these developments are in ultrathin channels up to 1 nm in diameter, it is reasonable to assume that the hydration of hydroxyapatite nanocrystals will lead to the effect of disjoining pressure. This, in our opinion, is the driving force, sliding walls ultrathin channels of collagen fibrils. Let us summarize the above considerations.

At osteoporosis, the bone matrix of the following events occurs

1. ultra-thin channels of collagen fibrils are expanding;
2. viscosity of water in the bone matrix increased;
3. mineral phase is exposed to amorphization and the thickness of hydroxyapatite nanocrystals increases;
4. the effect of disjoining pressure, sliding walls ultrathin channel develops.

All this causes a decrease in the rates of chemical reactions occurring in the aqueous phase of ultrathin channels of the general parts of collagen fibrils, some of whom lose the ability to catalyze the crystallization of hydroxyapatite. Giving this degree of osteoporosis will depend on the volume of the above changes in the nanoporous structure of the bone matrix.

## 5. Conclusion

As follows from the results of AFM and dynamic IR spectroscopy, in the early stages of osteoporosis, swelling of collagen fibrils develops, which indicates a decrease in the number of nanoporous structures. The consequence of this is the hydratation of HAP nanocrystals and their dismantling of the structures of collagen-apatite interfaces. One possible consequence of swelling of collagen fibrils is a depression in the spatial arrangement of hydroxyapatite nucleation sites located on the surfaces of protocollagen fibrils. In this situation, the collagen fibrils lose their catalytical properties and, accordingly, the rate of crystallization of HAP will be reduced. This is, in our opinion, the key mechanism for development of osteoporosis in the early stages of its development.

## Figures and Tables

**Table 1:** The structural and adhesion parameters of the healthy and osteoporotic bones.

Parameters	Healthy bones	Osteoporotic bones
OS-diameter, nm	848 (64)	391 (61)
Adhesion forces of main parts of collagen fibrils, nN	18,6 (35)	38,0 (60)
Adhesion forces of friable parts of collagen fibrils, nN	26,4 (35)	52,0 (63)
Monoclinic modification of HAP, vol.%	6,0 (1)	12,0 (1)
Amorphous phase of HAP, vol.%	20,0 (1)	36,0 (1)

Absolute uncertainties in last digits are given in parentheses.

**Table 2:** Parameters of absorption bands of healthy and osteoporotic bones.

Parameters	Healthy bones	Osteoporotic bones
Band center, cm^−1^	5000 *⋯* 5200	5200
Shift of band center with vacuum pumping, cm^−1^	200	0
Evacuation rate	Nonexponential	Exponential
